# Are Machine Learning Models Used to Represent Accelerometry Data Robust to Age Differences?

**DOI:** 10.1093/geroni/igab046.2880

**Published:** 2021-12-17

**Authors:** Todd Manini, Chen Bai, Amal Wanigatunga, Santiago Saldana, Ramon Casanova, Mamoun Mardini

**Affiliations:** 1 University of Florida, Gainesville, Florida, United States; 2 Johns Hopkins Bloomberg School of Public Health, Baltimore, Maryland, United States; 3 Wake Forest University, Winston-Salem, North Carolina, United States; 4 Wake Forest School of Medicine, Winston-Salem, North Carolina, United States

## Abstract

Regular and sufficient amounts of physical activity (PA) are significant in increasing health benefits and mitigating health risks. Given the growing popularity of wrist-worn devices across all age groups, a rigorous evaluation for recognizing hallmark measures of physical activities and estimating energy expenditure is needed to compare their accuracy across the lifespan. The goal of the study was to build machine learning models to recognizing the hallmark measures of PA and estimating energy expenditure (EE), and to test the hypothesis that model performance varies across age-group: young [20-50 years], middle (50-70 years], and old (70-89 years]. Participants (n = 253, 62% women, aged 20-89 years old) performed a battery of 33 daily activities in a standardized laboratory setting while wearing a portable metabolic unit to measure EE that was used to gauge metabolic intensity. Participants also wore a Tri-axial accelerometer on the right wrist. Results from random forests algorithm were quite accurate at recognizing PA type; the F1-Score range across age groups was: sedentary [0.955 – 0.973], locomotion [0.942 – 0.964], and lifestyle [0.913 – 0.949]. Recognizing PA intensity resulted in lower performance; the F1-Score range across age groups was: sedentary [0.919 – 0.947], light [0.813 – 0.828], and moderate [0.846–0.875]. The root mean square error range was [0.835–1.009] for the estimation of EE. The F1-Score range for recognizing individual PAs was [0.263–0.784]. In conclusion, machine learning models used to represent accelerometry data are robust to age differences and a generalizable approach might be sufficient to utilize in accelerometer-based wearables.

